# High‐Throughput Parallel Optofluidic 3D‐Imaging Flow Cytometry

**DOI:** 10.1002/smsc.202100126

**Published:** 2022-06-05

**Authors:** Masashi Ugawa, Sadao Ota

**Affiliations:** ^1^ Research Center for Advanced Science and Technology The University of Tokyo 4-6-1 Komaba, Meguro-ku Tokyo 153-8904 Japan

**Keywords:** acoustofluidic particle focusing, imaging flow cytometry, light-sheet microscopy

## Abstract

3D light‐sheet microscopy is a powerful tool to obtain content‐rich information of cell populations. However, the limited frame rate of high‐quantum‐efficiency cameras hinders its application in high‐throughput flow cytometry. Herein, a high‐throughput 3D‐imaging flow cytometry technique based on light‐sheet microscopy that can screen thousands of cells per second is introduced. This method enables fast, parallel optofluidic scanning of cells by performing 1D acoustofluidic focusing on multiple cells under wide‐field light‐sheet microscopy with a single objective lens. Multicolor 3D‐imaging flow cytometry with cell‐line samples at a record detection throughput of over 2000 cells s^−1^ is demonstrated. Furthermore, an unprecedentedly large‐scale 3D‐morphology‐based flow cytometric analysis at an order of 10^5^ cells is demonstrated. Results show that the system is able to capture subcellular structures even at high throughputs and obtain cellular information that is overlooked under 2D‐imaging cytometry.

## Graphical abstract

High‐throughput 3D‐imaging flow cytometry is realized by parallel light‐sheet imaging of cells flowed with 1D acoustofluidic focusing. With a maximum detection throughput of over 2000 cells s^−1^, the technology enables large‐scale (>10^5^ cells) 3D‐morphology‐based analysis for practical 3D‐imaging flow cytometry.

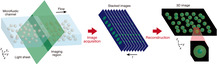

## 1 Introduction

Large‐scale analysis of cells is necessary for recognizing small subpopulations of cells and detecting rare cell events.^[^ ^1^, ^2^, ^3^ ^]^ For such purposes, fluorescence‐intensity‐based flow cytometry is routinely used because it can analyze 10^4^–10^6^ cells at the single‐cell level within several minutes.^[^ ^3^, ^4^ ^]^ However, such flow cytometric analysis becomes less effective if there are no designated markers to identify the cell populations of interest. For example, it has been challenging to decisively identify different stages of mitosis using flow cytometry,^[^ ^5^ ^]^ and instead, optical microscopy has been utilized to characterize these stages by observing morphologies of dividing cell nuclei.^[^ ^5^ ^]^ Still, compared to flow cytometry, optical microscopy has limited throughput and ability to perform large‐scale cell analysis, and therefore, finding statistically adequate number of cells in each mitotic stage, which are rare within the whole population, was difficult. Recently, imaging flow cytometry (iFCM) has been evolving to overcome the limitation of throughput and quantity of image analysis.^[^ ^6^, ^7^, ^8^, ^9^ ^]^ Nonetheless, most iFCM techniques are based on 2D images and are incapable of accurately resolving and understanding critical cellular structures and processes organized in three dimensions,^[^ ^10^, ^11^, ^12^, ^13^, ^14^, ^15^, ^16^ ^]^ including the morphology of dividing cell nuclei as well as biochemical pathways and colocalization events within cells.^[^ ^17^, ^18^ ^]^ Thus, high‐throughput 3D‐iFCM will be an indispensable tool for accurately characterizing large cell populations and consequently analyzing small and rare subpopulation of cells.

However, high‐throughput 3D‐iFCM is difficult compared to 2D‐iFCM because the extra dimension of 3D imaging increases the time needed to acquire an image. This is most evident in its analogy in microscopy. 3D imaging with conventional confocal scanning microscopy, where each point in space is sequentially acquired at a time, typically requires several minutes for image acquisition.^[^ ^19^, ^20^ ^]^ Moreover, although high sampling rates can be achieved by increasing the scan speed with acousto‐optic deflectors,^[^ ^21^ ^]^ the required high bandwidth limits the number of photons per sampling time^[^ ^22^ ^]^ resulting in low signal‐to‐noise ratio as the speed increases. In contrast to confocal microscopy, light‐sheet microscopy acquires each plane at a time and has enabled fast acquisition of high‐content 3D images of cells.^[^ ^14^, ^15^, ^16^, ^19^ ^]^ Light‐sheet microscopy can achieve a higher voxel‐per‐second acquisition rate than confocal scanning because the total pixel‐readout rates of high‐speed fluorescence cameras are faster than the bandwidths of single‐pixel fluorescence detectors due to the parallelization of pixels.^[^ ^23^, ^24^, ^25^, ^26^ ^]^ More importantly, because it acquires each plane at a time, compared to point scanning, the acquisition time of each unit voxel is longer, and therefore, a higher signal‐to‐noise ratio can be achieved, even with the same voxel‐per‐second acquisition rate.^[^ ^23^ ^]^

Despite such potentials, implementing light‐sheet microscopy to image cells flowing in a channel has been a challenge. In its typical configuration made of two objective lenses closely placed and directed at the sample—one for light‐sheet excitation and the other for detection—the cell flow had to be directed toward the detection objective for imaging a cross section of the flow. Consequently, the flow channel had to be bent to clear out from the spatial obstruction of the objective and required complex microchannel fabrication.^[^ ^27^, ^28^, ^29^ ^]^ With such channel design, if the cells were to be flowed at high throughput, a large quantity of cells will exist between the imaging region and the objective lens and will interfere the image formation due to scattering effects. Furthermore, the throughput was limited when the optical sectional image was acquired one cell at a time^[^ ^29^, ^30^, ^31^ ^]^ due to the limited frame rate of high‐quantum‐efficiency cameras, which are necessary for fluorescence imaging. Multiple cells can be imaged simultaneously in a microfluidic chip for assembled cells such as spheroids,^[^ ^30^, ^31^, ^32^ ^]^ but imaging multiple isolated cells was difficult. This is because the profile of flow velocities in microchannels and the contacts of cells with channel walls induce difference in cell velocity and cell rotation, thereby preventing stable translational motion of cells required for flow‐scan‐based imaging cytometers. Consequently, the throughput of light‐sheet‐microscopy‐based 3D‐iFCM was limited to less than 50 cells s^−1^.^[^ ^28^, ^29^, ^30^, ^31^ ^]^

In this work, we developed a high‐speed iFCM method capable of obtaining 3D cell images for detailed analysis of intracellular structures at a detection throughput of over 2000 cells s^−1^, which is the fastest to the best of our knowledge. This is achieved by using a fluorescence light‐sheet imaging technique called oblique‐plane microscopy^[^ ^33^, ^34^, ^35^ ^]^ (OPM) which allows us to image a plane ranging in the depth direction with a single objective lens, thus avoiding the spatial obstruction of high‐NA objectives for optofluidic light‐sheet imaging. Furthermore, we combine this imager with acoustic particle focusing^[^ ^36^, ^37^ ^]^ that confines the stream of cells within a semi‐1D rectangular region. This confinement allows us to narrow the acquisition region of a high‐speed sCMOS camera for maximizing its frame rate while providing a uniform flow velocity of the flowing cells and preventing their rotation, both of which are critical for obtaining an accurate 3D image with optofluidic scanning. With the above integration, we were able to achieve a throughput which is over an order of magnitude higher than previous light‐sheet‐microscopy‐based 3D‐iFCM. Furthermore, using our system, we were able to perform large‐scale 3D‐image‐based analysis of cells at an order of 10^5^ cells, which is two orders of magnitude larger than previous reports on 3D‐iFCM and becomes comparable to the scale of conventional fluorescence‐intensity‐based flow cytometry.

## 2 Results

### 2.1 Principles of 3D Light‐Sheet iFCM

**Figure 1:**
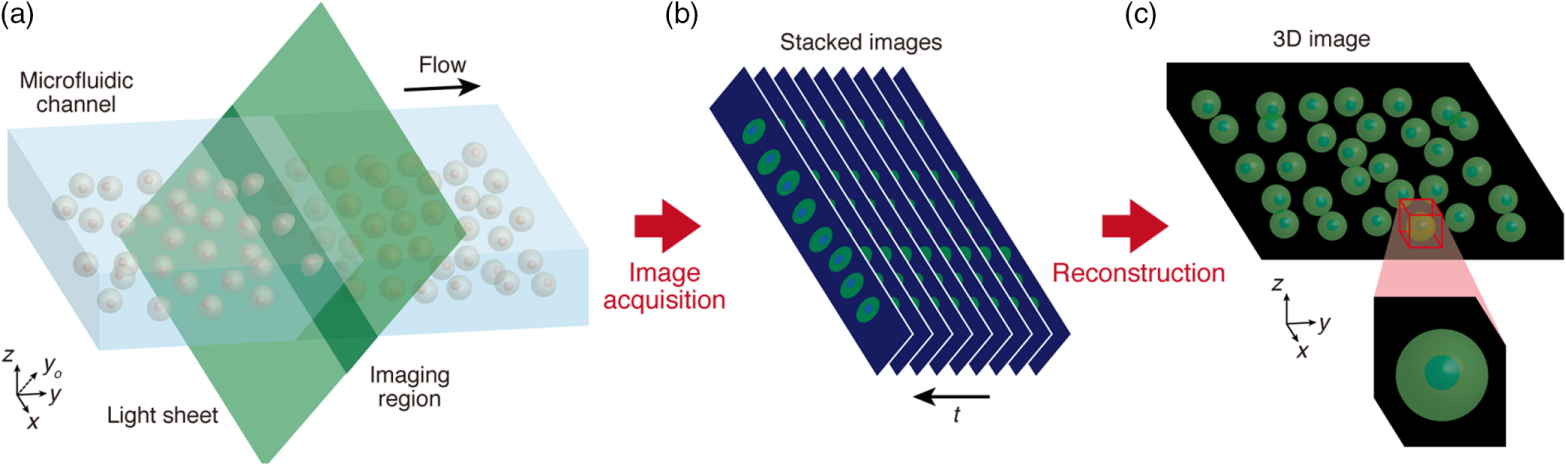
Schematic of high‐throughput parallel optofluidic 3D‐iFCM. a) Image acquisition of multiple cells synchronously flowing in a microfluidic channel by optofluidic light‐sheet imaging. The cells flowing at a constant velocity in the *y*‐direction are imaged at a region along the *xy*_o_‐plane (oblique plane) where the channel intersects with the excitation light sheet. b) Diagonal stacked images corresponding to the optically sectioned images of the cells obtained by continuous acquisition at the static imaging region in (a) when the cells are moving in the *y*‐direction. Time *t* in (b) corresponds to the –*y‐*direction in (a). c) 3D image of the flowing cells reconstructed from the diagonally stacked images in (b).

Our 3D light‐sheet iFCM works as depicted in **Figure** **1**. Cells are allowed to flow in the *y*‐axis direction at a constant velocity in parallel to each other while aligned at the center of the channel in the *z*‐axis direction (Figure 1a). Then by using OPM, we obtain images of the channel cross section at a plane that diagonally intersects with the channel (*xy*_o_‐plane in Figure 1a). By continuously acquiring the image of a stationary oblique plane, we are able to scan the 3D volume of the translating cells (Figure 1b) and consequently retrieve their 3D image (Figure 1c).

**Figure 2:**
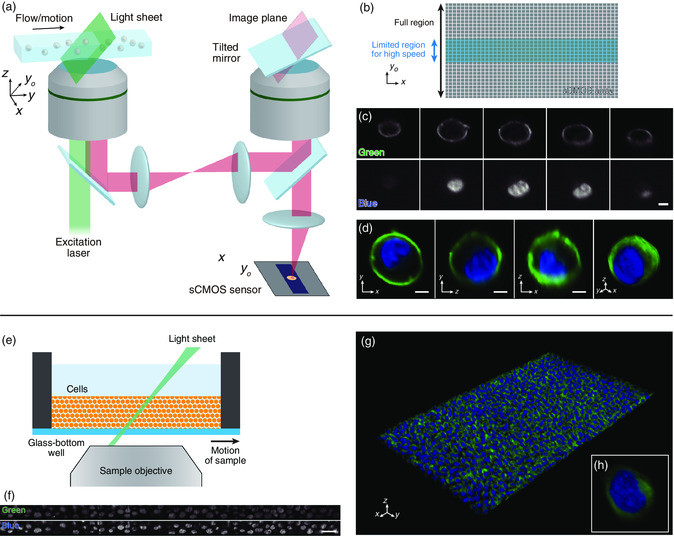
Cellular imaging with OPM using a single sample objective lens. a) Optical setup for OPM. The sample is placed on top of the sample objective on the left. The remote objective on the right is used along with the tilted mirror to convert the image from the oblique plane to the lateral plane. This converted image is then projected onto a sCMOS sensor. b) Illustration of sCMOS pixel region. To achieve a high frame rate, the active area on the sCMOS array is limited in the *y*_o_‐direction. On the other hand, the active area in the *x*‐direction is not limited as it does not affect the frame rate. c) Sequential cross‐sectional images for a single cell. Top panels correspond to green fluorescence (CellMask Green) and the bottom panels correspond to blue fluorescence (DAPI). Scale bar = 5 μm (horizontal). d) Overlayed cross‐sectional images and 3D reconstructed image of the same cell as (c). From left to right: *xy*‐plane cross section, *zy*‐plane cross section, *xz*‐plane cross section, and isometric projection of a 3D image. e) Schematic for high‐throughput imaging of cells in a glass‐bottom well. The well is moved in the lateral direction for scanning the sample. f) Example oblique‐plane image for high‐throughput imaging of K562 cells deposited in a glass‐bottom well. The top and bottom panels correspond to the green fluorescence (CFSE) and blue fluorescence (DAPI), respectively. Scale bar = 30 μm (horizontal). g) 3D reconstructed image from high‐throughput imaging of K562 cells deposited in a glass‐bottom well. Green and blue colors correspond to fluorescence from CFSE and DAPI, respectively. h) Close‐up of a single cell in (g).

The optical system used to perform OPM is illustrated in **Figure** **2**a (see also Figure S1, Supporting Information, for detailed principle). Here, we used a remote objective lens and a tilted mirror which enable imaging of an oblique plane instead of a conventional lateral image plane.^[^ ^38^, ^39^ ^]^ The oblique‐plane image at the sample objective, which is illuminated by a light sheet, is transferred to the remote objective. This image is then reflected by the tilted mirror where the image orientation is changed to the lateral plane, so that it can be imaged with an arrayed detector such as a commercial high‐speed sCMOS camera. In our setup, we use two excitation lasers and two sCMOS cameras to obtain two different colors of fluorescent images simultaneously (Figure S2, Supporting Information).

To achieve high‐throughput imaging, we operate the sCMOS by limiting the rows on the sensor array used for acquisition (Figure 2b). This is because sCMOS cameras operate by reading each row at a time, and therefore, by limiting the number of rows used during acquisition, they can run at a faster frame rate. On the other hand, the number of columns on the sensor used during acquisition does not affect the frame rate. Therefore, to maximize the number of cells that can be captured in a single frame, it is most efficient to image a region that is long in one dimension and narrow in the other dimension. Here, we use an active pixel region of 128 × 2560 pixels to achieve a frame rate of over 700 frames s^−1^.

**Figure 3:**
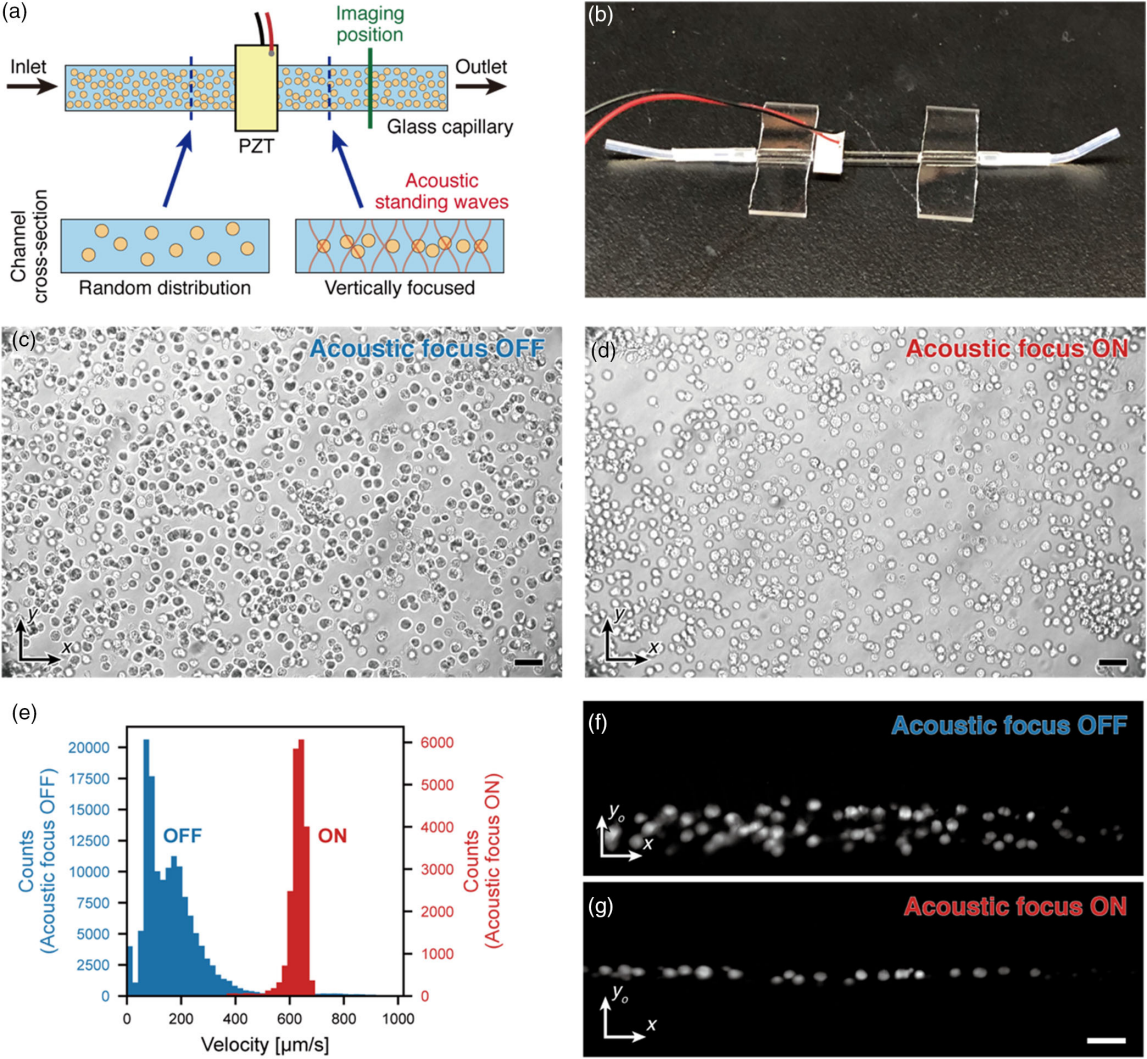
Acoustic‐focusing microfluidic device. a) Illustration of acoustic‐focusing microfluidic device which is fabricated by adhering a PZT onto a wide glass capillary. When the PZT is turned on at a certain range of frequency, acoustic standing waves are generated in the vertical direction of the channel and the cells are focused at the node while they flow through the position of the PZT. b) Photograph of the actual fabricated microfluidic device. c,d) Bright‐field images of cells in the lateral plane when the PZT is off (c) and on (d). When the PZT is off, the cells flow in different focus positions which appear on the image as different contrasts (d). When the PZT is on, the cells flow in the same focus position and appear as similar bright‐looking objects (d). Scale bar = 50 μm in (c) and (d). e) Velocity histogram of flowing cells when the PZT is off (blue) and on (red). The mean and the standard deviation for each distribution are 171 ± 126 and 626 ± 53 μm s^−1^, respectively. f,g) Fluorescence images of the oblique plane when the PZT is off (f) and on (g). When the PZT is off, the cells flow through the bottom half of the channel (f), but when the PZT is on, the cells flow through the vertical center of the channel (g). Scale bar = 50 μm (horizontal).

To perform optofluidic 3D imaging with the sCMOS at this high‐speed mode, it is necessary for the cells to flow through a narrow area at the cross section of the channel which corresponds to the narrow active region of the camera. We achieve this by using an acoustic‐focusing microfluidic device comprising a glass capillary with a wide rectangular channel and a piezoelectric transducer (PZT), as shown in **Figure** **3**a,b. The PZT generates acoustic standing waves in the vertical direction and confines the streams of cells inside a vertically narrow region in the horizontally wide capillary which coincides with the field‐of‐view (FOV) of the camera (Figure 3a). Moreover, this confinement allows the cells to flow at a constant velocity and without rotation, which is required to perform cell scanning with a stationary light sheet. This occurs because, when the channel width is substantially longer than the channel height, the shearing effect from the wall is dominant in the vertical direction and the parabolic flow velocity profile is present in only the vertical direction except near the side walls.^[^ ^39^ ^]^ Therefore, by vertically confining the cells with acoustic focusing, the flow velocity becomes uniform over a wide range of the channel.

### 2.2 Cell Imaging with OPM

To observe the imaging capability of our microscope, we first obtained a 3D image of a single cell with its subcellular components stained. K562 chronic myelogenous leukemia cells with the cell membranes stained with CellBrite Green (Biotium) and the nuclei stained with 4′,6‐diamidino‐2‐phenylindole (DAPI) were placed on a glass slide. This glass slide was moved in the lateral direction at a velocity of 5 μm s^−1^ with a motorized stage to obtain sequential cross sections of the oblique plane at a frame rate of 20 frames s^−1^ (Figure 2c, Movie S1, Supporting Information). Using about 100 of such frames, a 3D image of a single cell was reconstructed (Figure 2d, Movie S2, Supporting Information). From the obtained images, it can be seen that the structures of the cell membrane and the nucleus are clearly distinguishable and subcellular features can be resolved. For example, not only the outer cell membrane, but the nuclear membrane can also be seen in the green fluorescence images, and the localization of chromosomes in the nucleus can be observed in the blue fluorescence images. The advantage of 3D imaging is this ability to spatially resolve subcellular structures in all directions.

To confirm the high‐throughput imaging ability of our system, we further demonstrated wide‐field 3D imaging of cells on a glass‐bottom well. A suspension of K562 cells stained with carboxyfluorescein succinimidyl ester (CFSE) and DAPI was deposited into a glass‐bottom well plate in which the cells were settled to the bottom of the well. This well plate was moved in the lateral direction at a velocity of 0.5 mm s^−1^ with a motorized stage to obtain sequential cross sections of the cells in the oblique plane at a frame rate of 720 frames s^−1^ (Figure 2e,f, Movie S3, Supporting Information). Using 600 frames, a 3D image of the cell population in a region of 724 × 433 × 38 μm was reconstructed (Figure 2g, Movie S4, Supporting Information). From this 3D image, using a cell‐segmentation algorithm (see Experimental Section), we counted 2682 cells obtained in an acquisition time of 833 ms, corresponding to an experimentally measured detection throughput of 3220 cells s^−1^. Such high throughput can be achieved because cells overlapping in the depth direction can be clearly resolved as in the cross‐sectional images (Figure 2f). Even at such high throughput, our system is able to capture subcellular features (Figure 2h) which is desirable for high‐content screening of cells.

### 2.3 Parallel Flowing of Cells with Acoustic Focusing

To confirm the fluidic control obtained by our acoustic focusing microfluidic device, we compared the flowing position and the velocity of cells when the PZT was turned on and off. We used a 200 μm × 2 mm glass capillary for the channel and applied a 4.01 MHz sine‐wave signal to the PZT. A suspension of K562 cells stained with CFSE and DAPI with a concentration of about 1 × 10^7^ cells mL^−1^ was allowed to flow through the device at a flow rate of 10 μL min^−1^. According to images with conventional bright‐field imaging in the lateral plane, when the PZT was off, the cells appeared to flow at different focus positions and at different velocities (Figure 3c, Movie S5, Supporting Information). Furthermore, some cells appeared to rotate while translating through the FOV (Movie S5, Supporting Information). When the PZT was turned on, the cells appeared to flow at the same focus position and velocity, and the cells did not appear to rotate (Figure 3d, Movie S5, Supporting Information). Additionally, to quantitatively justify the effect of the PZT, we performed particle velocity analysis based on fluorescence images in the lateral plane (see Experimental Section). Results in Figure 3e show that the velocity profile had a wide distribution with a coefficient of variance (CV) of 0.74 when the PZT was off, but the velocity profile had a narrow distribution with a CV of 0.09 when the PZT was on.

We further investigated the focusing effect with our OPM system. Because our imaging system can observe the channel cross section directly, we can accurately obtain the vertical (*z*‐axis) position of where the cells are flowing. In this experiment, we imaged the oblique plane at 20 frames s^−1^ with an active pixel region of 800 × 2560 pixels, covering the whole vertical dimension of the channel. When the PZT was off, the cells flowed at the bottom side of the channel due to gravity (Figure 3f, Movie S6, Supporting Information). In contrast, when the PZT is turned on, the cells flowed and aligned as a tight line at the vertical center of the channel (Figure 3g, Movie S6, Supporting Information). It can be further observed that, in the former case, the retention time of the cells was longer as the cells were closer to the bottom indicating that the flow velocity was slower at the bottom of the channel (Movie S6, Supporting Information). In the latter case, because the cells were farther from the channel wall, the retention time of the cells was shorter (Movie S6, Supporting Information). These results are consistent with velocity profiles obtained with numerical simulations.^[^ ^8^, ^40^, ^41^ ^]^

### 2.4 High‐Throughput Parallel 3D‐iFCM

**Figure 4:**
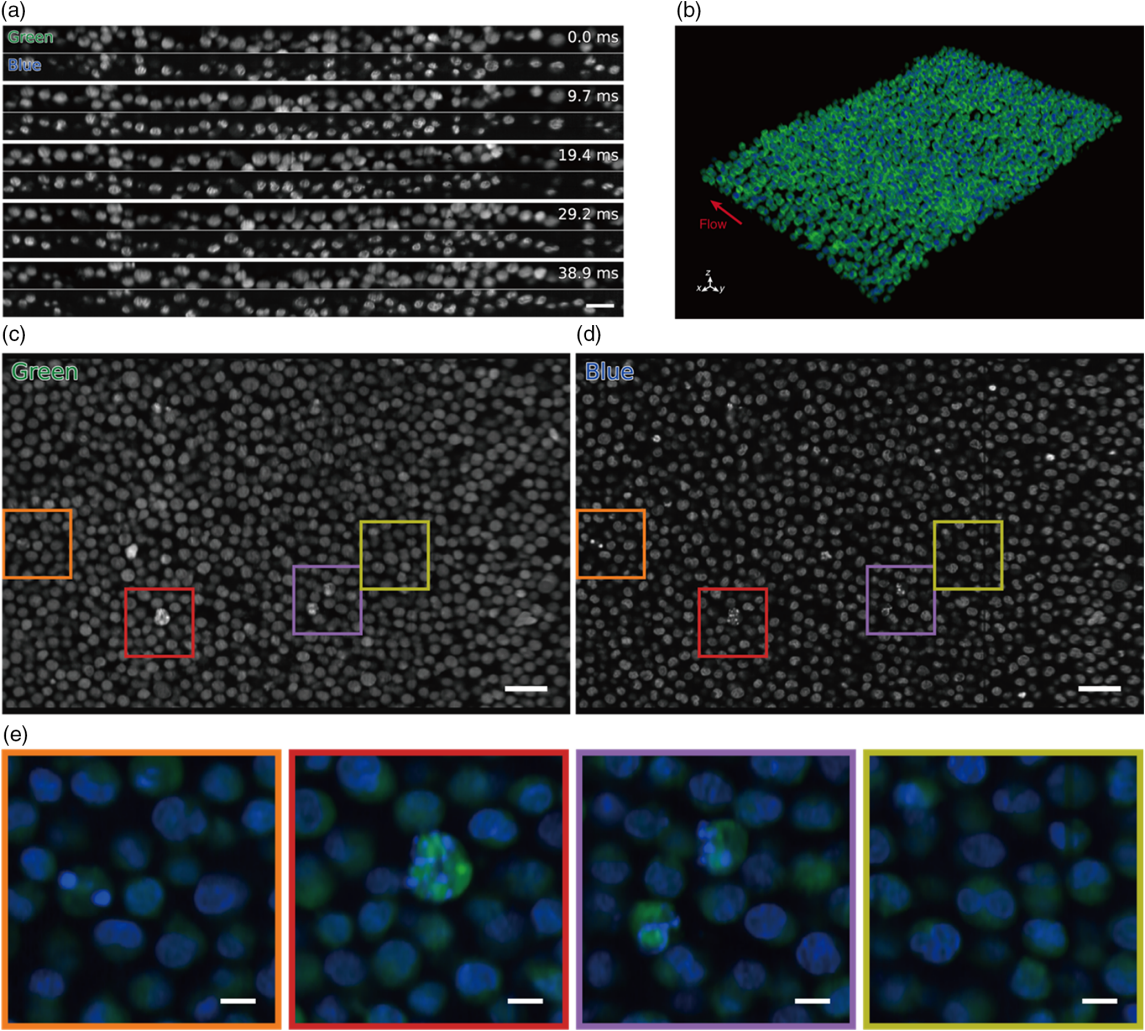
High‐throughput 3D‐iFCM. a) Green (CFSE) and blue (DAPI) fluorescence images of K562 cells in the oblique plane obtained at different time frames. Scale bar = 30 μm (horizontal). b) Overlayed isometric projection of a 3D‐reconstructed image of CFSE (green) and DAPI (blue) stained K562 cell population taken in 833 ms. The direction of flow is in the –*y*‐axis direction. c,d) *xy*‐cross sections of the K562 cell population in (b). Scale bar = 50 μm. e) Overlayed close‐up view of cells in each of the square regions in (c) and (d). Colors of the frames in (e) correspond to the colors of the squares in (c) and (d). Scale bar = 5 μm.

By applying OPM to cells flowing in the acoustic focusing device, we performed high‐throughput 3D‐iFCM. A suspension of K562 cells stained with CFSE and DAPI with a concentration of about 1 × 10^7^ cells mL^−1^ was allowed to flow through the device at a flow rate of 10 μL min^−1^. Obtained raw cross‐sectional images taken at a frame rate of 720 frames s^−1^ are shown in **Figure** **4**a and Movie S7, Supporting Information. Because of the vertical acoustic focusing, the cells flowing through the channel with a height of 200 μm are confined within the narrow FOV of the sCMOS corresponding to a height of 38 μm. The flow velocity of the cells was measured to be 0.53 mm s^−1^. From 600 frames, a 3D image of the cells flowing was reconstructed (Figure 4b, Movie S8 and S9, Supporting Information). In this 3D image, 1927 cells were counted at 833 ms, corresponding to an experimentally measured detected throughput of 2312 cells s^−1^ (Figure S8, Supporting Information). It can be seen that we are able to obtain images of cells using flow with similar quality to those obtained using a motorized stage and that we can observe the detailed structure of the cell nuclei clearly (Figure 4c–e).

**Figure 5:**
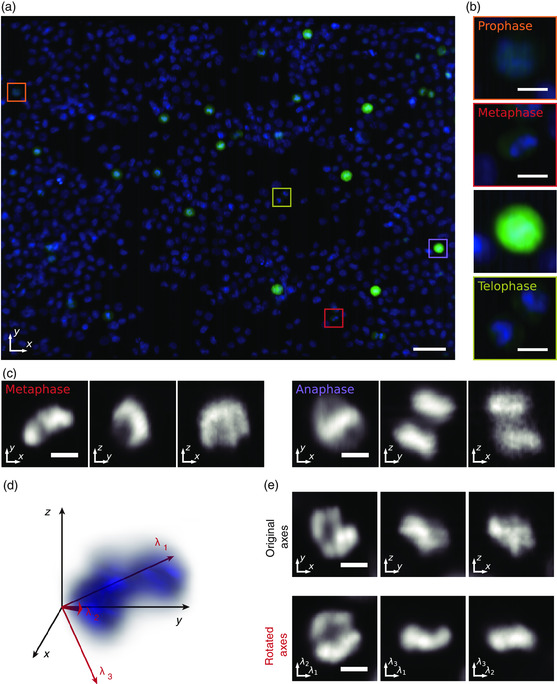
Mitotic phase analysis with high‐throughput 3D‐iFCM. a) Overlayed *xy*‐projected view of a whole population of K562 cells stained with MPM‐2‐AlexaFluor 488 (green) and DAPI (blue) in a single acquisition taken in 857 ms. Scale bar = 50 μm. b) Close‐up view of cells in the square regions in (a). Each cell was defined to be in prophase, metaphase, anaphase, and telophase (top to bottom). c) 2D‐projected views of blue fluorescence (DAPI) from different directions for the metaphase (left panels) and anaphase (right panels) cells in (b). From left to right in each set of panels: projection onto the *xy*‐, *yz*‐, and *xz*‐planes. It can be seen that the separation of chromosomes is evident from the *yz*‐ and *xz*‐planes for the anaphase cell but difficult to recognize from the *xy*‐plane. d) 3D image of a DAPI‐stained region in a metaphase cell. *x*‐, *y*‐, and *z*‐axes show the original axes and *λ*_1_–*λ*_3_ are the new axes used for rotation which are based on the eigenvectors obtained in principal component analysis (see Experimental Section). The unit vectors for *λ*_1_–*λ*_3_ are [–0.54, 0.82, 0.20], [0.71, 0.31, 0.63], and [0.45, 0.48, –0.75], respectively, in the original Cartesian coordinate. A rotating 3D image is available in Movie S10, Supporting Information. e) 2D‐projected views of (d) in the original axes (top panels) and rotated axes (bottom panels). The metaphase plane for this cell is difficult to distinguish in the original axes but is evident in the rotated axes. Scale bar = 5 μm in (b), (c), and (e).

Being able to screen the morphology of the cell nuclei, we further applied this system to perform mitotic phase analysis. A suspension of K562 cells stained with antimitotic protein MPM‐2 and DAPI with a concentration of about 1 × 10^7^ cells mL^−1^ was allowed to flow through the device at a flow rate of 10 μL min^−1^. From the 3D image acquired in a total acquisition time of 857 ms (**Figure** **5**a), 1248 cells were counted. Within this population, we defined 60 cells MPM‐2 positive. Then from further analysis with DAPI‐stained images, we defined 34, 10, and 2 cells to be in prophase, metaphase, and anaphase, respectively, and 2 pairs of cells to be in telophase (Figure 5b and S9, Supporting Information). Such analysis of cell cycle phases within mitosis using nuclear staining can be inaccurate with 2D imaging because the metaphase plane or the splitting of chromosomes may not be apparent depending on the orientation of the cell. For example, the separation of the chromosomes in anaphase for the cell in Figure 5c cannot be observed in the conventional lateral plane (*xy‐*plane) images. In contrast, with our method, we were able to resolve the positions of the chromosomes in the *z*‐direction and distinguish that this cell was in anaphase (Figure 5c).

Furthermore, we found that for some cells, observations in the original *xy*‐, *xz*‐, or *yz*‐planes were not sufficient to interpret how the chromosomes are aligned for identifying cell phases. As an example, for the DAPI‐stained region in Figure 5d, when examined from these planes, it is difficult to identify the alignment of the chromosomes (Figure 5e, top panels). To reveal the structure, we obtained the long and short axes of the DAPI‐stained region using principal component analysis (PCA, see Experimental Section) and observed it in the rotated axes. When the view was rotated, the alignment of chromosomes was easily identified (Figure 5e, bottom panels). Such analysis cannot be performed with 2D or multiple‐angle 2D views and is only possible with 3D imaging.

**Figure 6:**
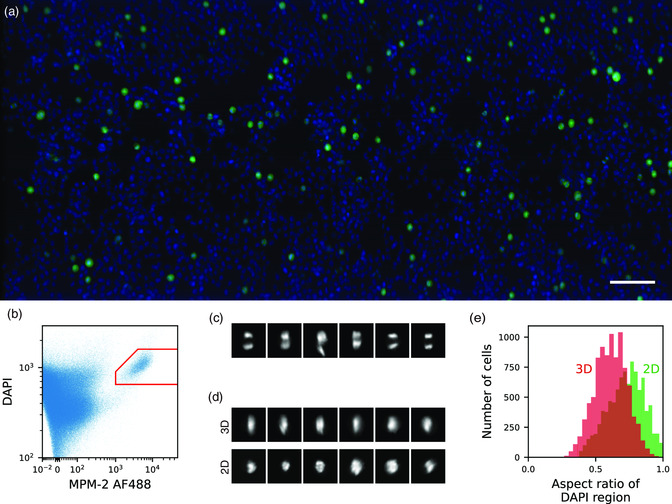
Large‐scale 3D‐image analysis with high‐throughput 3D‐iFCM. a) Overlayed *xy*‐projected view of 1% of the whole imaged population of K562 cells stained with MPM‐2‐AlexaFluor 488 (green) and DAPI (blue). Scale bar = 100 μm. b) Scatter plot of MPM‐2‐AlexaFluor 488 and DAPI intensities obtained from the 3D image. The red region shows the gated region in which the cells were defined MPM‐2 positive. 10 814 cells were within this gate. c) 2D‐projected view of cells with two DAPI‐stained regions. d) Comparison of 2D‐projected view for 3D and 2D imaging of cells with a single DAPI‐stained region. For the top row, the direction of projection is rotated so that the longest and shortest axis becomes the vertical and horizontal directions, respectively. For the bottom row, a *xy*‐projected view was obtained from the 3D image to simulate an image from 2D‐iFCM. e) Histogram of the aspect ratio of DAPI‐stained region obtained from 3D and 2D images of cells with a single DAPI‐stained region. 2D images tend to overestimate the aspect ratio as can be confirmed from images in (d).

Finally, we performed large‐scale 3D‐image analysis of flowing cells at the order of 10^5^ cells. Using K562 cells stained with antimitotic protein MPM‐2 and DAPI, we obtained 3D images of this cell population in flow. From these images, we detected 408 937 cells in a total acquisition time of 286 s (**Figure** **6**a) and performed the following analysis. First, the intensities of MPM‐2 and DAPI for each of the cells were obtained to gate MPM‐2 positive cells (Figure 6b). Within the positive cells, we counted the number of DAPI‐stained regions inside the cells to distinguish cells in anaphase or telophase (Figure 6c). Then for the cells with only a single DAPI‐stained region, we obtained its aspect ratio from the 3D image and compared it to the aspect ratio that would be obtained for a 2D image (Figure 6d,e). It can be seen from the histogram that 2D imaging overestimates the aspect ratio compared to 3D imaging due to the loss of a dimension. Therefore, to accurately obtain structural profiles of cells, 3D imaging at such high throughput is necessary.

## 3 Discussion

The high throughput of our 3D‐iFCM is achieved by utilizing the fact that cells are not occluded, and therefore, are spatially resolved in 3D space. In 2D‐iFCM, two or more cells can overlap each other in the obtained image depending on their positions and, as a result, cannot be resolved. In most cases, this problem is dealt with by diluting the cell concentration so that the cells are less likely to coincide and by rejecting the data derived from multiple cells, limiting the throughput of analyzing cells in practice. However, with our 3D‐iFCM, we do not have to consider this problem, and therefore, we are able to flow the cells at a high concentration to achieve a high throughput.

The throughput of our imaging system is governed by the resolution of the image and the frame rate of the camera. Higher resolution will reduce the FOV resulting in fewer cells captured in the same frame. Here, we use a 20× objective lens with an NA of 0.75, which has sufficient resolution for various cell analysis such as classifying cell types or stages in cell cycle with iFCM.^[^ ^42^ ^]^ As for the frame rate, the sCMOS sensor we use on the camera (Zyla 5.5, Andor) is currently among the fastest with high quantum efficiency to capture fluorescence images. In the demonstrations we presented, we use a global shutter configuration of the sCMOS, but we can further enhance the frame rate by a factor of 2 by using a rolling shutter because the top and bottom halves of the sCMOS can work in parallel. With this configuration, it is possible to double the throughput, but spatial distortion caused by the nonsimultaneous acquisition by each pixel‐line has to be compensated to obtain a 3D image. Because the main limitation of throughput arises from the frame rate of the camera, future improvements in camera‐sensor technology will directly lead to higher throughputs.

## 4 Conclusion

In conclusion, we have demonstrated high‐throughput parallel 3D‐iFCM which records a detection throughput of 2312 cells s^−1^ and performed large‐scale 3D‐image analysis at the order of 10^5^ cells. Our method provides fast, large‐scale screening of 3D cell structures which is necessary for accurate analysis of cells at the single‐cell level. Because of the massive multidimensional data that our method acquires, we anticipate that, further combined with machine learning techniques, it will give us new insights on cellular biology and enable various biotechnological applications in the near future.

## 5 Experimental Section

5.1

5.1.1

##### Optical Setup

Two 20× objective lenses (UPLSAPO20X, Olympus) were used each for the sample objective and the remote objective. The image in the sample objective was transferred to the remote objective with two tube lenses. This image was reflected by the tilted mirror with which the orientation was changed. Using a beam splitter, the reflected image was guided toward a dichroic mirror (Chroma) where it was split into green and blue images. These images were each imaged by a sCMOS camera (Zyla 5.5, Andor Technology) with a corresponding bandpass filter (Chroma). For the excitation, a 405 nm laser (Stradus 405‐250, Vortran) and a 473 nm laser (gem 473, Laser Quantum) were shaped into light sheets with Powell lenses (Laserline Optics Canada) and cylindrical lenses (Thorlabs) and combined with a dichroic mirror (Chroma). The combined lasers were inserted into the system with a multiband dichroic mirror (Semrock) and guided toward the sample so that it coincides with the imaging plane. Samples were put on a linear motorized stage (OptoSigma) which was controlled with a microstep controller (SHOT‐702, OptoSigma). The lasers and cameras were operated so that the excitation and the exposure for all colors were performed simultaneously. See also Figure S2, Supporting Information, for detailed setup. All oblique‐plane images have different horizontal and vertical scales due to the difference in refractive index at the sample (water) and at the tilted mirror (air). The obtained image data are deposited in BioImage Archive.^[^ ^42^ ^]^

##### Microchannel Fabrication

The microfluidic device used to flow cells with acoustic focusing comprises mainly a glass capillary and a piezoelectric transducer (PZT) attached to it as illustrated in Figure 2a. The glass capillary with an inner height of 200 μm, an inner width of 2 mm, and a wall thickness of 0.14 mm (3520, VitroCom) was connected to fluorinated ethylene‐propylene tubes with heat‐shrink tubings. Fluorinated ethylene‐propylene tubes were used to prevent cell aggregation within the tubings. The channel was treated with 1% Pluronic F‐68 (Gibco) or 0.5% Pluronic F‐127 (Biotium) before flowing the sample to prevent cells from attaching to the channel wall. A PZT with a resonance frequency of 3.75 MHz (3.75Z5 × 10R‐SYX (C‐213), Fuji Ceramics) was used. During the operation of the PZT, a sine‐wave signal with a peak‐to‐peak amplitude of 5.0 V was applied. The cell suspension was allowed to flow through the microfluidic device using a syringe pump (Harvard Apparatus).

##### Sample Preparation

To demonstrate 3D cell imaging, we used fluorescent‐stained K562 chronic myelogenous leukemia cells (JCRB). K562 cells were cultured in RPMI1640 (#R8758, Sigma) with fetal bovine serum (F7524, Sigma) and Antibiotic‐Antimycotic (#15 240 062, Gibco).

For the imaging of a single cell, K562 cells were first fixed with 2% paraformaldehyde (PFA) for 15 min at room temperature. Then they were stained with 1/200 concentration CellBrite Green (Biotium) for 5 min at room temperature and subsequently stained with 50 μg mL^−1^ DAPI (Dojindo) overnight. The cells were resuspended in PBS and were loaded on a glass chamber slide with a cover‐glass thickness of 0.2 mm (MUR‐500, Matsunami).

For high‐throughput imaging on a motorized stage, K562 cells were stained with 10 μg mL^−1^ CFSE (Dojindo) for 30 min at 37 °C, then fixed with 2% PFA for 15 min at room temperature, and subsequently stained with 50 μg mL^−1^ DAPI overnight. The cells were resuspended in PBS and passed through a 40 μm‐pore cell strainer. Then the suspension was added to a glass‐bottom well plate with a glass thickness of 0.17 mm, and the cells were allowed to settle to the bottom of the well.

For high‐throughput imaging in a microfluidic device, K562 cells were stained with CFSE, then fixed with 2% paraformaldehyde for 15 min at room temperature, and subsequently stained with DAPI overnight. The cells were resuspended in IsoFlow Sheath Fluid (Beckman Coulter) and were passed through a 40 μm‐pore cell strainer before loading.

For the mitotic phase analysis, K562 cells were first fixed with 2% paraformaldehyde for 15 min at room temperature. Then they were pretreated with 1 × Permeabilization Buffer (eBioscience) for 10 min and then treated with 1/500 concentration antimitotic protein MPM‐2 (ab14581, Abcam, Lot: GR3290238‐1) in 1 × Permeabilization Buffer for 1 h, followed with 1/250 concentration Goat Anti‐Mouse IgG AlexaFluor 488 (ab150113, Abcam, Lot: GR3284150‐1) in 1 × Permeabilization Buffer for 1 h. After washing with 1 × Permeabilization Buffer, the cells were finally stained with 5 μg mL^−1^ DAPI overnight. The cells were resuspended in IsoFlow Sheath Fluid and were passed through a 40 μm‐pore cell strainer before loading. For the large‐scale analysis, the same procedures were followed except that the lot # of the antimitotic protein MPM‐2 was changed to GR3364338‐1.

##### Image Processing and Analysis

Image processing and analysis were performed with custom code written in Python 3. For cell segmentation, a watershed algorithm^[^ ^43^ ^]^ using mahotas^[^ ^44^ ^]^ was used (Figure S6a and S8a, Supporting Information). The cell segmentation and the cell counting were validated by obtaining the 3D area of the cells and comparing the cell count with the ratio of the area sum and the area median (Figure S8b, Supporting Information). Codes are available on Zenodo.^[^ ^45^ ^]^

##### Image‐Based Particle Velocity Analysis of Flowing Cells

Particle velocity analysis was performed with CFSE‐stained K562 cells with a concentration of 1.5 × 10^6^ cells mL^−1^. Fluorescence images were obtained on an inverted microscope (Ti‐U, Nikon) with a 10× objective lens (CFI Plan Fluor 10X, Nikon), GFP filter set (Chroma), and a CMOS camera operating at 44 frames per second (ASI1600MM Pro, ZWO). Particle analysis was performed using Trackpy (v0.4.2).^[^ ^46^ ^]^ First, particles in all frames were detected, as in shown Figure S7, Supporting Information. Then the detected particles in each frame were linked with particles detected in consecutive frames. Finally, the velocity of each particle in each frame was calculated from the displacement in neighboring frames to obtain the histogram in Figure 3e. Codes are available on Zenodo.^[^ ^45^ ^]^

##### Image‐Based Mitotic Phase Analysis with MPM‐2 and DAPI

The 3D image of K562 cells stained with MPM‐2‐AlexaFluor 488 and DAPI was first put through cell segmentation. Then MPM‐2 positive cells were obtained from the fluorescence intensities of each cell (Figure 6b and S9a, Supporting Information). Within these positive cells, cells that were clipped and were difficult to identify the cell phase were omitted. From the remaining mitotic cells, the anaphase cells were distinguished by the number of spots, and the telophase cells were distinguished by combinations of two adjacent mitotic cells. Cells in prophase and metaphase were distinguished by the long and short axes ratio.

The long and short axes were obtained by PCA of the DAPI‐stained region. The first and third components (or eigenvectors) of PCA were assigned as the long and short axes, respectively. Figure S10 and S11, Supporting Information, show the blue fluorescence of MPM‐2 positive cells in the orientation in which the long and short axes are the vertical and horizontal axes, respectively.

For the large‐scale analysis, 20 consecutive acquisitions of 10 000 frames were used. The reconstructed 3D images were put through cell segmentation, and then the intensities of MPM‐2 and DAPI were obtained. From a scatter plot of MPM‐2 and DAPI intensities, the cells in the gate in Figure 6 were further analyzed. For each of the gated cells, the longest and shortest axes were determined by PCA, and the aspect ratio was obtained by dividing the shortest axis by the mean of the other two axes.

All codes are available on Zenodo.^[^ ^45^ ^]^

## Conflict of Interest

Both authors have filed patent applications related to the high‐throughput parallel optofluidic 3D‐imaging flow cytometry.

## Supporting information

Supplementary Material

## Data Availability

Data and codes are available on BioStudies (https://www.ebi.ac.uk/biostudies/studies/S‐BIAD219) and Zenodo (doi:10.5281/zenodo.5739323).
