# Supplementary material for: High‐Throughput Parallel Optofluidic 3D‐Imaging Flow Cytometry
Source: Small Sci. 2022 Jun 5;2(7):2100126. doi: 10.1002/smsc.202100126 (PMC11935936; doi:10.1002/smsc.202100126)
Supplement: Supplementary file 1 — Supplementary Material [file SMSC-2-2100126-s001.zip › 3D-cyto_supp_v10_clean.pdf]

Supporting Information

High-throughput parallel optofluidic 3D-imaging flow cytometry

*Masashi Ugawa and Sadao Ota**

Compensation of index mismatch

Because the sample is in water (n = 1.33) and the tilted mirror at the remote objective is in air (n = 1.0), the intermediate image at the objective lens becomes shortened in the depth (*z*-axis) direction. This amount can be calculated with the following equation.[1]

$$\frac{d'}{d} = \frac{1}{100} \cdot \sum_{k=1}^{100} \frac{\tan\left(\sin^{-1}\frac{k\mathrm{NA}}{100n_1}\right)}{\tan\left(\sin^{-1}\frac{k\mathrm{NA}}{100n_2}\right)}$$

When NA = 0.75, $d'/d = 1.412$, and this is used for the reconstruction of the 3D image. This value is validated in Figure S3g.

Effect of PZT on the microfluidic device and image

The acoustofluidic focusing occurs due to the radiation force and not the acoustic wave itself.[2] The actual amount of vibration or displacement that the acoustic wave causes can be estimated by the displacement of the PZT and theoretical calculations. According to the specification of the PZT,[3] the displacement u of the PZT for material C213 can be estimated by

$$u = V \cdot d_{33}$$

where V is the input voltage and $d_{33}$is the piezoelectric constant which is 310 × 10^{-12} V•m/N. Therefore, when the input voltage is 3.0 V as in the demonstrations, the displacement becomes 0.93 nm. Note that this value is when the PZT is vibrating alone, and when attached to a device, it would be much smaller. Furthermore, in previous reports, the displacement and deformation of an acoutofluidic device was simulated to be about ± 0.4 nm.[4] Thus, the displacement that the acoustic wave causes is negligible compared to the optical resolution and does not affect the imaging quality.

PCA for obtaining long and short axes of a specified region

PCA, according to its definition, derives the vector which direction has the maximum variance as the vector of coefficient for the first principal component.[5] To obtain such unit vector $\boldsymbol{\alpha}_1$, the covariance matrix $\boldsymbol{\Sigma}$ of all the points in the region is used. When $\boldsymbol{\alpha}_1$ maximizes the variance, $\boldsymbol{\alpha}_1^{\mathrm{T}}\boldsymbol{\Sigma}\boldsymbol{\alpha}_1$ becomes maximum. Therefore, because $\boldsymbol{\alpha}_1$ is a unit vector (i.e. $\boldsymbol{\alpha}_1^{\mathrm{T}}\boldsymbol{\alpha}_1 = 1$), using a Lagrange multiplier λ,

$$\boldsymbol{\alpha}_1^{\mathrm{T}}\boldsymbol{\Sigma}\boldsymbol{\alpha}_1 - \lambda(\boldsymbol{\alpha}_1^{\mathrm{T}}\boldsymbol{\alpha}_1 - 1)$$

is to be maximized. Differentiation with respect to $\boldsymbol{\alpha}_1$ gives

$$(\boldsymbol{\Sigma} - \lambda\mathbf{I})\boldsymbol{\alpha}_1 = \mathbf{0}$$

and the quantity to be maximized is

$$\boldsymbol{\alpha}_1^{\mathrm{T}}\boldsymbol{\Sigma}\boldsymbol{\alpha}_1 = \lambda$$

This shows that the vector of coefficient $\boldsymbol{\alpha}_1$ for the first principal component is the eigenvector corresponding to the largest eigenvalue of $\boldsymbol{\Sigma}$. Similarly, it can be shown that the vector which direction has the minimum variance is the vector of coefficient for the last principal component, i.e. the eigenvector corresponding to the smallest eigenvalue of $\boldsymbol{\Sigma}$. When

this is adopted to a specified region in 3D space, the long and short axis of the region can be obtained.

Comparison of PCA and other methods for obtaining long and short axes of a specified region

When PCA that adopts singular value decomposition[6] was used the calculation time for obtaining the long and short axes of a single DAPI region was 4.1 s for 100 loops. On the other hand, conventional methods such as ellipsoid fitting and rectangular fitting took 4.1 s and 10.8 s, respectively, for a single loop (codes available on Zenodo). Furthermore, the latter two methods do not seem to fit properly because only the points at the edges are taken into account for fitting. The points inside of the region can be used for better fitting, but this will increase the time required for calculation.

Preparation of alginate hydrogel beads

The fluorescent alginate hydrogel beads in Figure S3g were prepared according to the following procedures. 1% sodium alginate (IL-6, Kimica corporation) solution in 50 mL of pH 5.5, 2-(N-morpholino)ethanesulfonic acid (MES) buffer (100 mM MES, 300 mM NaCl pH 5.5) with 3.85 mg (20 μmol) of 1-ethyl-3-(3-dimethylaminopropyl)carbodiimide (EDC) and 20 μg of Alexa Fluor 488 hydrazide (Invitrogen) was stirred overnight. 2-propanol was added to this solution to precipitate the alginate. The precipitate was redissolved with distilled water and reprecipitated with 2-propanol. The final precipitate was allowed to dry to obtain Alexa Fluor 488 alginate. A 2% solution of this fluorescent alginate was mixed with 2% of non-fluorescence sodium alginate and ethylenediaminetetraacetic acid (EDTA)-Ca buffer (50 mM CaCl2, 50 mM EDTA) at a ratio of 15:35:50. Using this solution, alginate solution droplets were created in Droplet Generation Oil for EvaGreen (Bio-Rad) with a droplet generating microfluidic device fabricated with PDMS. Finally, the created droplets were gelled with 0.05% acetic acid, and the gel beads were taken out of the oil by washing with 20% 1H,1H,2H,2H-perfluoro-1-octanol in Novec 7200 (3M) to obtain fluorescent alginate hydrogel beads.

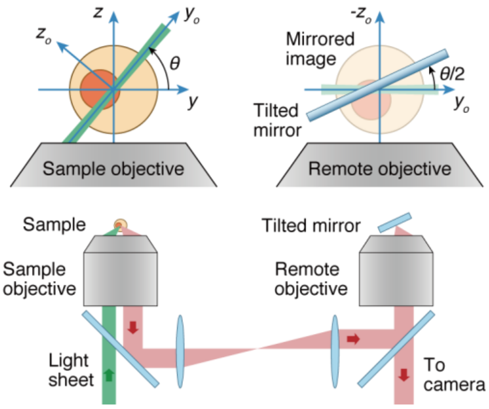

Figure. S1. Principle of oblique-plane microscopy. The image in the illuminated plane along the y_o-axis at the sample objective is transferred to the remote objective. Using a tilted mirror, the image is reflected so that the y_o-axis at the sample objective becomes perpendicular to the optical axis in the remote objective.

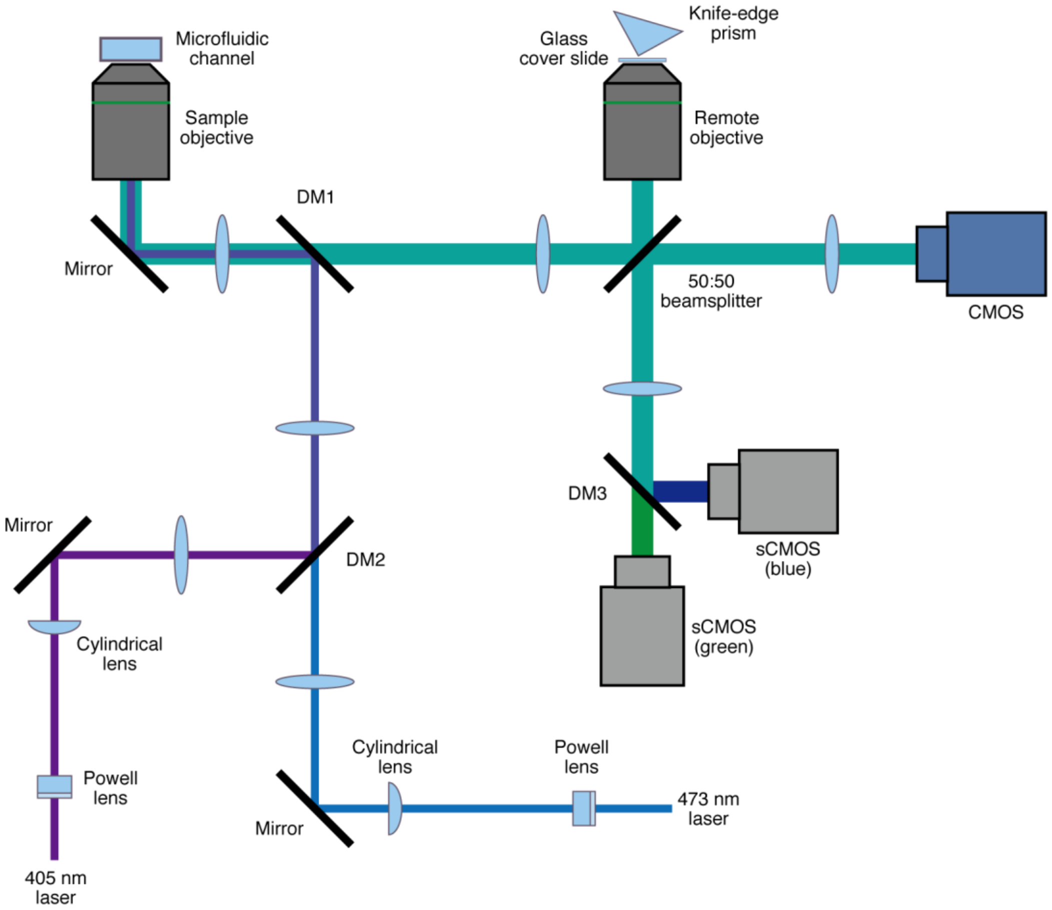

Figure S2. Detailed optical setup. All lenses are achromatic spherical lenses from Thorlabs unless otherwise noted. The oblique-plane image at the microfluidic channel on the sample objective is transferred using two lenses to the remote objective. On the remote objective, the oblique-plane image is converted to a lateral-plane image using a knife-edge prism (Thorlabs) as a tilted mirror. A glass cover slide with a thickness of 0.15 mm (Matsunami) is inserted between the knife-edge prism and the remote objective to compensate the cover-glass correction of the objective. The reflected image from the knife-edge prism is separated from the input image using a 50:50 beamsplitter and guided towards the sCMOS cameras. A dichroic mirror (DM3) splits the image into two colors and each are imaged onto a sCMOS camera with a corresponding optical bandpass filter (green: 525 nm center wavelength, 50 nm bandwidth; blue: 440 nm center wavelength, 40 nm bandwidth). On the other end of the 50:50 beamsplitter, an CMOS camera is placed to observe the lateral-plane image at the sample objective. A 473 nm laser and a 405 nm laser are used as the excitation lasers. Each is passed through a Powell lens to elongate the beam in the vertical direction (perpendicular to the figure) and through a cylindrical lens to focus in the horizontal direction. The two wavelengths are combined with a dichroic mirror (DM2), is inserted into the detection setup using another dichroic mirror (DM1), and is guided into the sample objective to coincide with the imaging oblique plane.

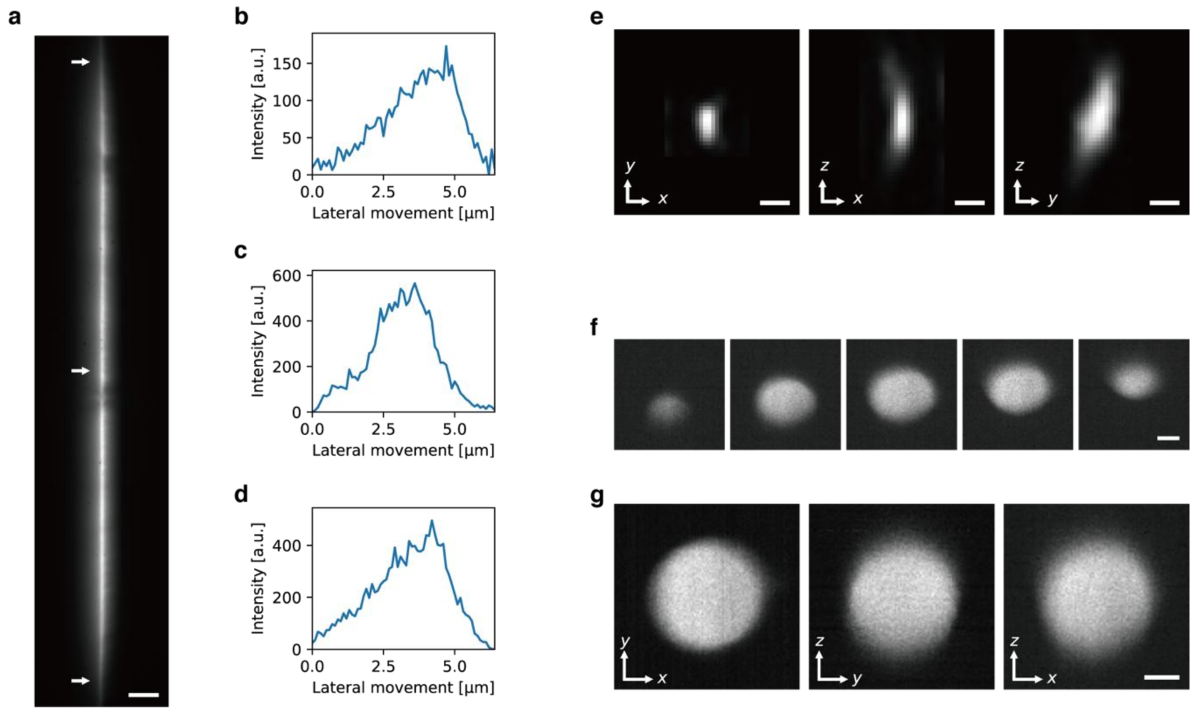

Figure S3. Characterization of oblique-plane microscopy system. a) Image of light sheet taken with a glass chamber slide with fluorescent solution. (a) shows the image in the lateral plane (*xy*-plane in Figure 2a). b,c,d) Profile of light sheet at different positions corresponding to the arrows in (a). (b): top arrow; (c): middle arrow; (d): bottom arrow. The profiles were each obtained by measuring the intensity of a laterally translated 100-nm fluorescent bead (F8803, Invitrogen) on a cover glass. The FWHM were 2.6, 2.2, 2.8 µm for (b), (c), and (d), respectively. e) PSF obtained by imaging a 100-nm fluorescent bead (F8803, Invitrogen) in 1% agarose gel inside a glass chamber slide (MUR-500, Matsunami). The image was taken from the cover-glass side of the chamber slide while translating it on a motorized stage at a velocity of 1 µm/s. The diagonal PSF resembles the theoretical PSF previously reported.[7,8] The FWHM in the *x*-, *y*-, and *z*-axes directions were 0.57, 1.1, and 2.0 µm, respectively. Because the size of a single pixel on the camera corresponds to 0.286 µm at the image plane, it can be considered that the *x*-axis resolution is limited to twice the pixel size according to the Nyquist criterion. f,g) Raw oblique-plane images (f) and cross-sectional images of 3D reconstructed image (g) of an alginate hydrogel microsphere. The FWHM in the *x*-, *y*-, and *z*-axes directions were 16.0, 16.3, 15.7 µm, respectively. This shows that the compensation of index mismatch is correct. Scale bars: 30 µm for (a), 1 µm for (e), and 5 µm for horizontal direction of (f) and (g).

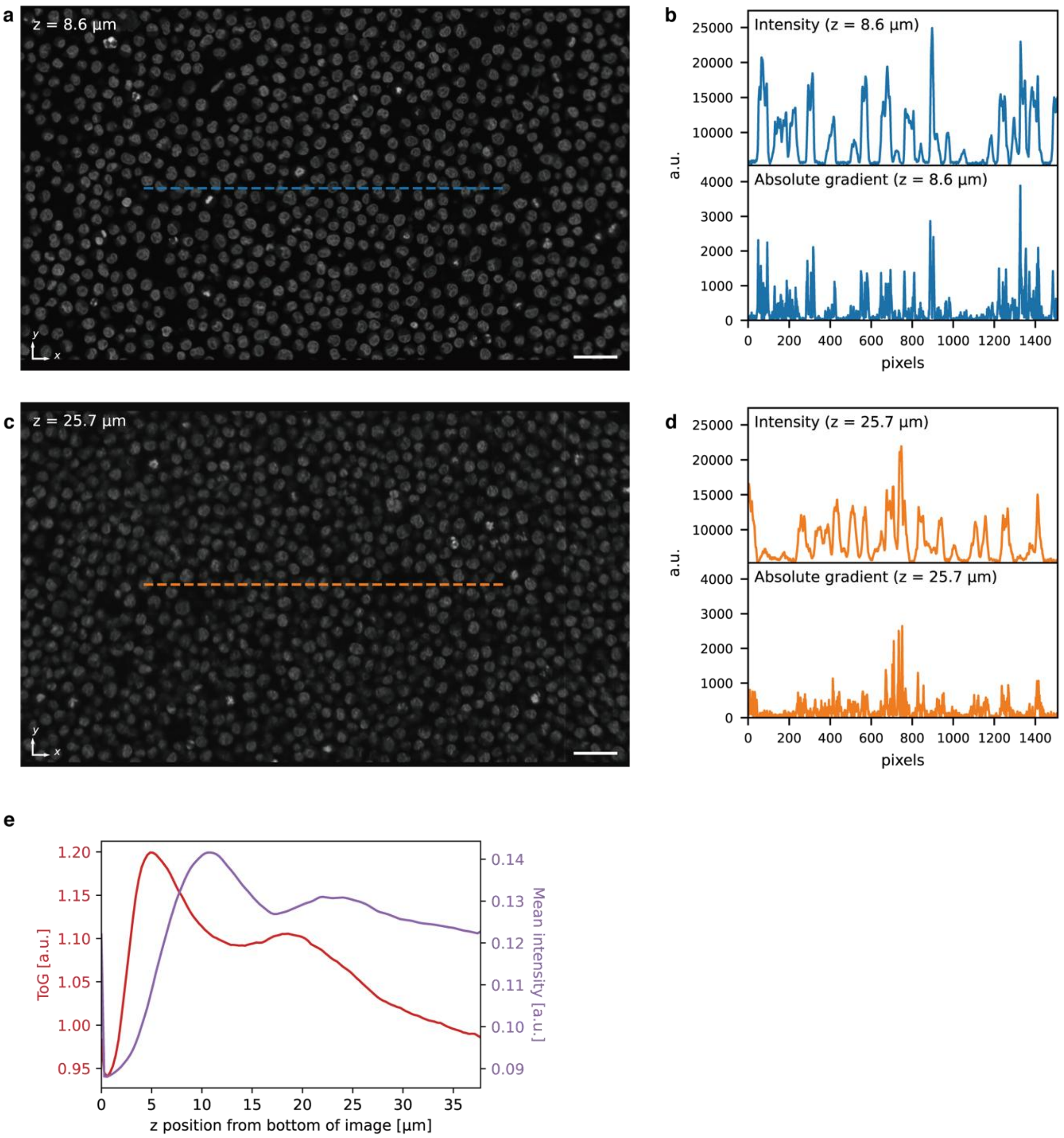

Figure S4. Comparison of different *z*-axis positions for the 3D image in Figure 2g. a) *xy* cross section of 3D DAPI image at 8.6 µm from the bottom of the image. b) Intensity profile (top panel) of the dashed line in (a) and its absolute gradient (bottom panel). c) *xy* cross section of 3D DAPI image at 25.7 µm from the bottom of the image. It can be seen that the images of the cells are less sharp compared to (a). d) Intensity profile (top panel) of the dashed line in (b) and its absolute gradient (bottom panel). The absolute gradient becomes smaller than (b) which shows that the image in (c) is less sharp than (a). e) Tamura coefficient of gradient modulus (ToG) and mean intensity for each *xy* cross section of 3D DAPI image for the image. ToG is used in holography as a criterion for autofocusing.[9] ToG drops as the *z* position of the *xy* cross section becomes further from the bottom of the image. This shows that DAPI image is less focused at deeper cross sections due to the scattering of the cells and the width of the light sheet. Because ToG is also affected by the sparsity of the image, its values at lower z-axis positions (close to 0 µm) are small because there are only small number of cells visible in the image and the image is sparse (as can be seen by the mean intensity). Scale bars = 50 µm for (a) and (c).

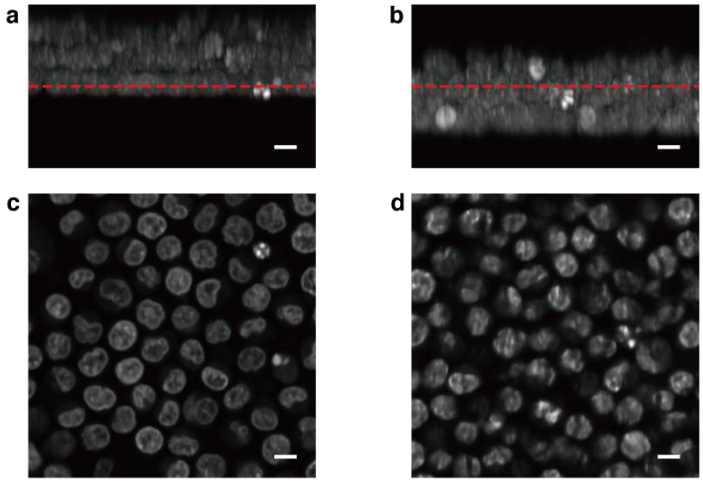

Figure S5. Effect of cell scattering on image formation when observing cells deposited in a glass bottom well. a,b) Max projection of *xz* cross section. (a) is taken when the bottom layer of the cells is at the center of the imaging region while (b) is taken when the bottom layer of the cells is at a lower position than (a). c,d) *xy* cross sections of the 3D image corresponding to the *z*-axis position of the red dashed lines in (a) and (b), respectively. The image in (c) shows clear shapes of the nuclei while the image in (d) shows the nuclei are out of shape. This is due to the scattering effects of the cells that lie underneath (i.e. cells between the layer and the objective lens). Scale bars = 10 µm.

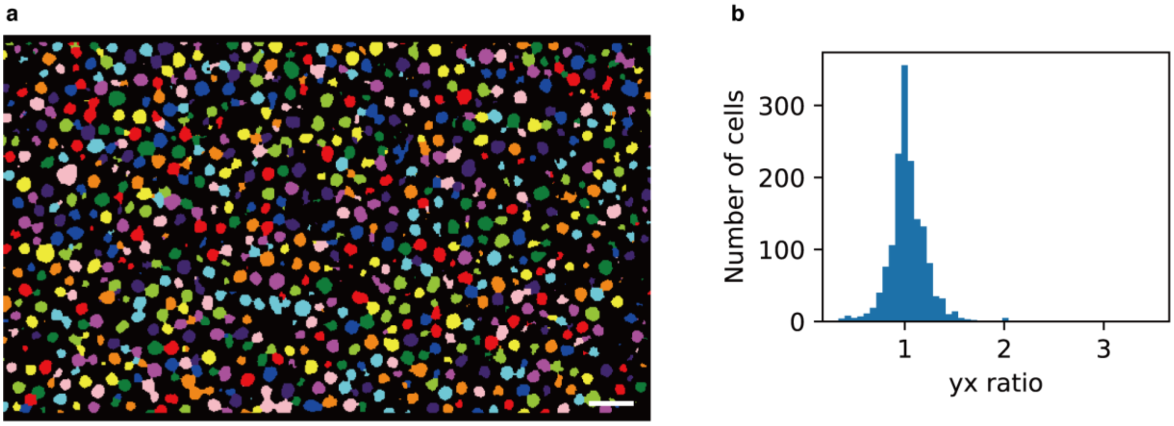

Figure S6. Cell aspect ratio analysis for high-throughput 3D imaging. a) Cross-sectional view of the segmentation result of the 3D image obtained in Figure 2g. Different colors show the region of different cells. Because only a limited number of colors is used, the same color is label multiple cells. Total of 2,682 regions were segmented. b) Aspect ratio of the *y*-axis direction and *x*-axis direction of the segmented regions of the 3D image obtained in Figure 2g. The mean and the standard deviation were 1.04 ± 0.20. From this result, it can be inferred that the mean aspect ratio of the cells is close to 1.0 and this can be used to calculate the flow velocity from the acquired images by oblique-plane microscopy. Scale bar = 50 µm.

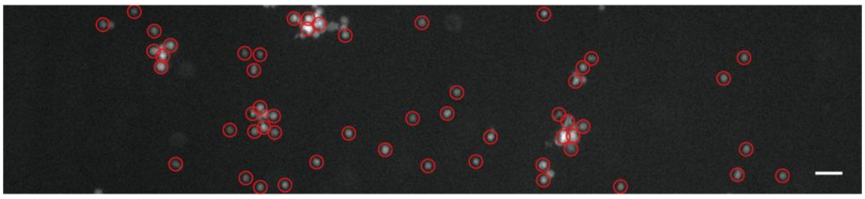

Figure S7. Example frame in particle velocity analysis for flowing cells. Single frame from a fluorescence movie of flowing cells. Cells are flowing from top to bottom. Red circles indicate particles that were detected using Trackpy in this frame. Some cells are not detected due to clustering and were not used to measure the velocity. Scale bar = 50 µm.

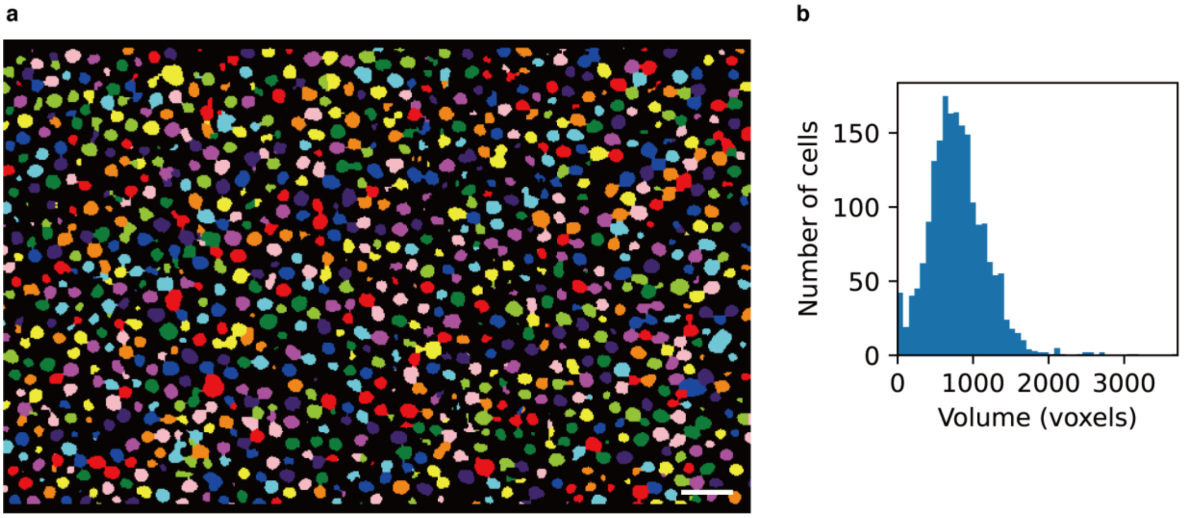

Figure S8. Validation of segmentation and cell count. a) Cross-sectional view of the segmentation result of the 3D image obtained in Figure 4. Different colors show the region of different cells. Because only a limited number of colors is used, the same color is label multiple cells. Total of 1,927 regions were segmented, which where we derived the cell count and the detection throughput of 2,312 cells/s from. When the regions which touch the boundary of the whole image are removed, the total count will be 1,413. b) Histogram of the cell volume derived from the segmentation in (a). The median volume is 760 voxels. The sum of all the volumes (corresponding to the total voxels of the binarized region before segmentation) divided by the median volume of the cells equals 2,020, which is in close agreement to the cell count. Therefore, although there are segmentation errors and cell clipping at the edges, the used algorithm is adequate for performing cell counting from a 3D image of a large population of cells. Scale bar = 50 µm.

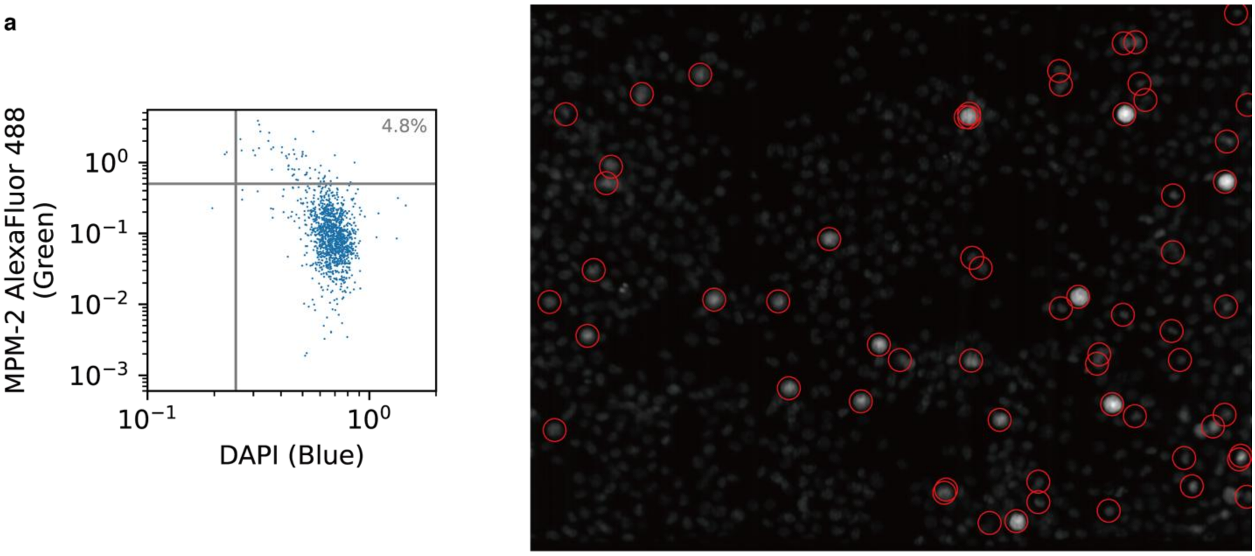

Figure S9. Fluorescence intensity analysis of MPM-2-AlexaFluor 488 and DAPI stained K562 cells. a) Fluorescence histogram of DAPI vs MPM-2-AlexaFluor 488 intensity used for analyzing cell cycle. The intensity of MPM-2-AlexaFluor 488 was compensated for the spillover of DAPI. Both the intensity of DAPI and the intensity of MPM-2-AlexaFluor 488 were used to perform gating to obtain MPM-2 positive cells. 60 cells, corresponding to 4.8% of the population, were counted as positive. b) *xy*-plane projection of the intensity of green fluorescence (MPM-2-AlexaFluor488). Here, the intensity is not compensated for the spillover of DAPI. Red circles indicate cells that were counted positive by (a).

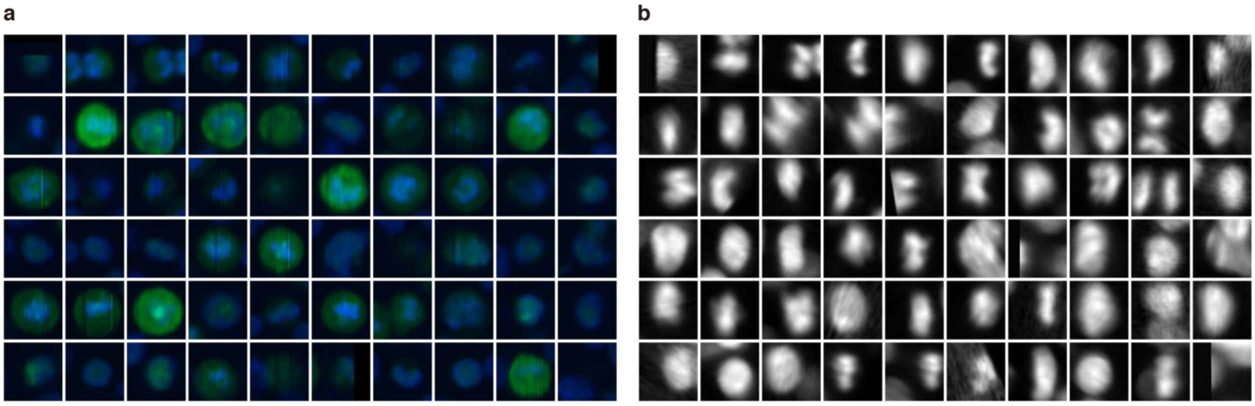

Figure S10. Images of MPM-2-AlexaFluor 488 positive K562 cells. a) Close up view of MPM-2-AlexaFluor 488 positive cells defined as in Figure S4. Green (MPM-2-AlexaFluor 488) and blue (DAPI) fluorescence are overlaid. b) Rotated view of MPM-2-AlexaFluor 488 positive cells defined as in Figure S4. Only the blue (DAPI) fluorescence is shown. The vertical and horizontal axes are the long and short axes of the DAPI-stained region obtained by principal component analysis (see Experimental Section). In (a) and (b), some images are clipped because the cell is close to the boundary of the entire imaging region, and some images are from the same cell due to segmentation errors.

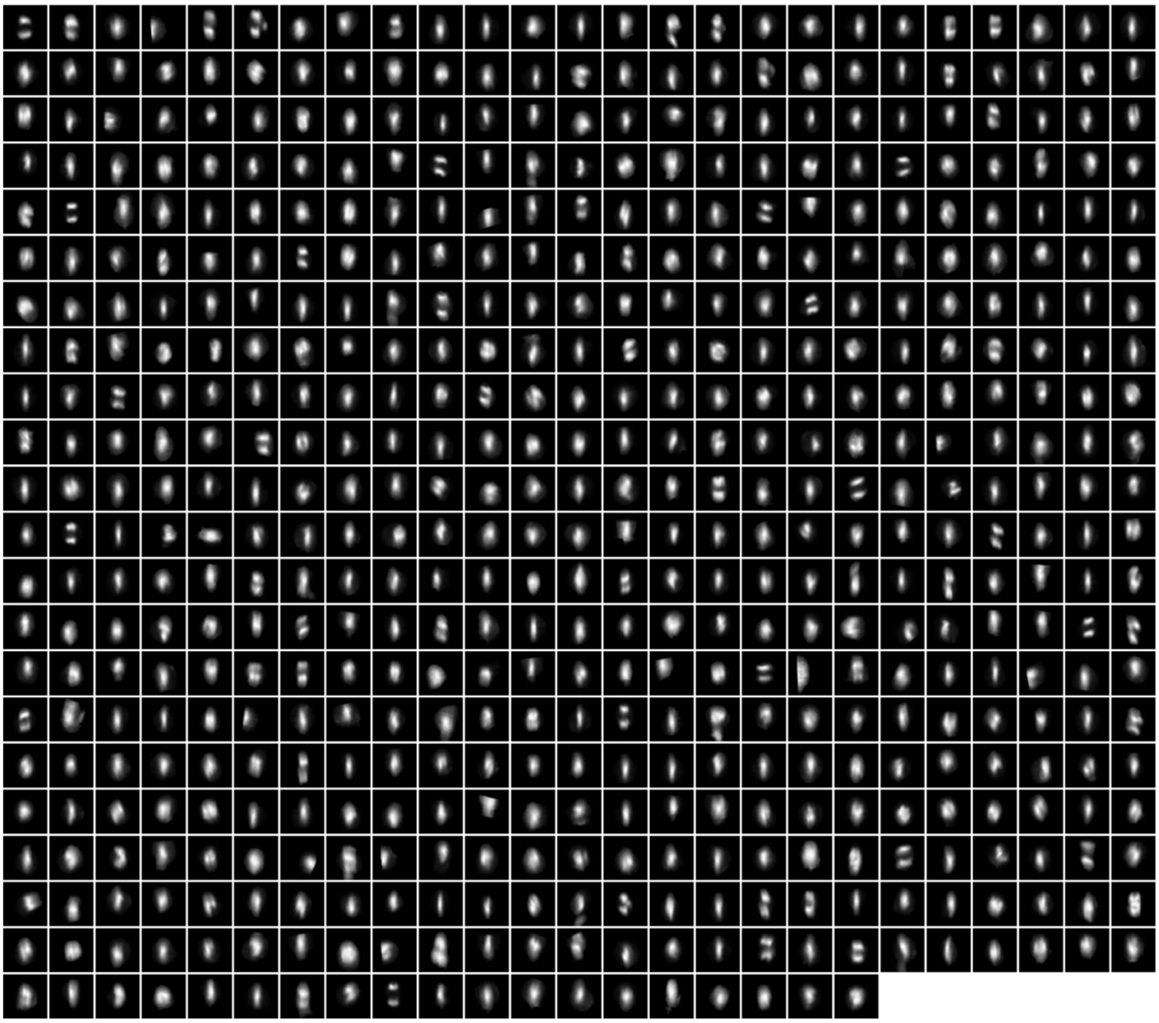

Figure S11. Rotated images of MPM-2-AlexaFluor 488 positive K562 cells for large-scale 3D-image analysis. Rotated view of MPM-2-AlexaFluor 488 positive cells defined as in Figure 6b for a single acquisition. The blue (DAPI) fluorescence regions of 544 cells are shown. The vertical and horizontal axes are the long and short axes of the DAPI-stained region obtained by principal component analysis (see Experimental Section). Some images are clipped because the cell is close to the boundary of the entire imaging region. The whole data is available on Zenodo.[10]

Supplemental Movie 1. Oblique-plane images of K562 cell stained with CellBrite Green and DAPI. Left: Green fluorescence (CellBrite Green). Right: Blue fluorescence (DAPI).

Supplemental Movie 2. Rotating 3D reconstructed image of K562 cell stained with CellBrite Green and DAPI. Green: CellBrite Green. Blue: DAPI.

Supplemental Movie 3. Oblique-plane images of K562 cell population scanned with a motorized stage. Top: Green fluorescence (CFSE). Bottom: Blue fluorescence (DAPI).

Supplemental Movie 4. Rotating 3D reconstructed image of K562 cell population scanned with a motorized stage. Green: CellBrite Green. Blue: DAPI.

Supplemental Movie 5. Bright field images of flowing K562 cells when the PZT is off (left) and on (right).

Supplemental Movie 6. Cross-sectional images of flowing K562 cells when the PZT is off (top) and on (bottom), taken with oblique-plane microscopy.

Supplemental Movie 7. Oblique-plane images of K562 cell population flowing in a microfluidic channel. Top: Green fluorescence (CFSE). Bottom: Blue fluorescence (DAPI).

Supplemental Movie 8. Rotating 3D reconstructed image of K562 cell population flowing in a microfluidic channel. Green: CellBrite Green. Blue: DAPI.

Supplemental Movie 9. Example z-stack image of K562 cell population flowing in a microfluidic channel. Green: CellBrite Green. Blue: DAPI.

Supplemental Movie 10. Rotating 3D reconstructed image of a DAPI stained region of a single K562 cell in metaphase.

References

[1] E. E. Diel, J. W. Lichtman, D. S. Richardson, *Nat. Protoc.* **2020**, *15*, 2773.
[2] H. Bruus, *Lab Chip* **2012**, *12*, 20.
[3] "Guidance to using piezoceramics," can be found under http://www.fujicera.co.jp/managed/wp-content/themes/fujicera/digitalbook/en/elements/, **n.d.**
[4] J. Dual, T. Schwarz, *Lab Chip* **2012**, *12*, 244.
[5] I. T. Jolliffe, *Principal Component Analysis*, Springer, New York, NY, **2010**.
[6] F. Pedregosa, G. Varoquaux, A. Gramfort, V. Michel, B. Thirion, O. Grisel, M. Blondel, P. Prettenhofer, R. Weiss, V. Dubourg, J. Vanderplas, A. Passos, D. Cournapeau, M. Brucher, M. Perrot, É. Duchesnay, *J. Mach. Learn. Res.* **2011**, *12*, 2825.
[7] J. Kim, T. Li, Y. Wang, X. Zhang, *Opt. Express* **2014**, *22*, 11140.
[8] J. Kim, M. Wojcik, Y. Wang, S. Moon, E. A. Zin, N. Marnani, Z. L. Newman, J. G. Flannery, K. Xu, X. Zhang, *Nat. Methods* **2019**, *16*, 853.
[9] Y. Zhang, H. Wang, Y. Wu, M. Tamamitsu, A. Ozcan, *Opt. Lett.* **2017**, *42*, 3824.
[10] M. Ugawa, S. Ota, *Zenodo* **2021**, DOI 10.5281/zenodo.5739323.
